# Facile Synthesis of IrCu Microspheres Based on Polyol Method and Study on Their Electro-Catalytic Performances to Oxygen Evolution Reaction

**DOI:** 10.3390/nano9081145

**Published:** 2019-08-10

**Authors:** Xuan Liu, Zichao Li, Luming Zhou, Kuankuan Wang, Xihui Zhao, Qun Li, Yujia Deng

**Affiliations:** 1 School of Chemistry and Chemical Engineering, Qingdao University, Qingdao 266071, China; 2 College of Life Sciences, Qingdao University, Qingdao 266071, China

**Keywords:** IrCu alloy, microspheres, polyol method, electrocatalytic, oxygen evolution reaction

## Abstract

The development of Ir-based catalyst with high efficiency for oxygen evolution reaction (OER) in acidic conditions is of great significance to the development of clean energy, but it still remains a significant challenge for shape controlled synthesis of Ir-based catalysts. This article presented a facile one-pot synthesis method that is based on polyol method for preparing IrCu microspheres. In the process of synthesis, formaldehyde solution and ethylene glycol were used as reducing agent and solvent, respectively, while poly(vinylpyrrolidone) was used as surfactant and dispersant, and all of them played important roles in the successful synthesis of Ir-Cu microspheres. The Ir-Cu microspheres, as synthesized, showed well sphere shape and smooth surface, while their alloy features were quite clear and the composition could be adjusted. Benefitting from the synergistic electronic effect between the Iridium and Cupric atoms from the alloy, the IrCu_0.77_ microspheres exhibited excellent electrocatalytic activity towards OER in 0.1 M HClO_4_ electrolyte, and to achieve 10 mA cm^−2^, IrCu_0.77_ microspheres only required the overpotential of 282 mV, which was much lower than that of commercial Ir/C catalysts.

## 1. Introduction

In recent decades, shape-controlled synthesis of Pt group metal nano/micro catalysts has been widely studied in the field of clean energy electrocatalysis due to their outstanding catalytic performance [1,2,3]. So far, researchers have successfully synthesized a variety of platinum, palladium-based nanocrystals with different morphologies, compositions, and sizes through various approaches (such as liquid phase chemical reduction, electrodeposition, hydrothermal solvothermal, etc.), and applied them in important electro-catalytic reactions, such as oxygen evolution reaction (OER), oxygen reduction reaction (ORR), and formic acid oxidation reaction (FAOR) [4,5,6,7,8,9,10,11,12,13,14,15,16]. As one of the platinum group noble metals, Iridium is an important and irreplaceable catalytic material, especially in the field of catalysis on OER, because of its outstanding catalytic activity and better stability than others, such as Ru-based catalysts; therefore, it is of great significance to the development of Ir-based catalysts [17,18,19,20,21].

In recent years, through the continuous efforts of researchers, the shape controlled synthesis of Ir-based nanocatalysts has made great progress [20,21,22,23,24,25,26,27,28,29,30,31,32,33,34]. Through shaped-controlled synthesis strategies, more and more Ir-based nanocrystals with different morphologies and different compositions have been reported, such as one-dimensional Ir-based nanowires, two-dimensional porous iridium nanosheets, Ir-based nanocrystals that are composed of basic crystal planes, such as (111), (100), (110), etc., which exhibited excellent catalytic activities and stabilities in electrocatalytic reactions, like OER [21,22,23,24,25,29,31,32,33,34,35,36]. In addition, the shape-controlled synthesis of Ir-based nanocatalysts by introducing other metals, such as transition metals Cu, Fe, Co, and Ni to form Ir-based alloy nanocatalysts has also drawn much attention of researchers due to the enhancements of the catalytic performance stemming from the synergistic electronic effect as well as the reduction of the cost [21,25,28,30,32,36,37,38]. Therefore, alloying Ir with transition metal could be considered to be a promising strategy to synthesize efficient Ir-based nano/micro catalysts. It is also worth mentioning that, from the bulk single crystalline plane to the nanoscale catalysts, the study of microscale Ir catalysts should be quite important and appealing, since it has been reported that the size of Pt-group catalysts could have significant effect on the catalytic performances besides the effect of crystalline facets [39,40].

Herein, for the first time, we reported a facile one-pot synthesis method that is based on polyol method for preparing IrCu microspheres. In the typical procedure of synthesis, Iridium (III) chloride and Copper (II) chloride dehydrate were the metal precursors, ethylene glycol, and formaldehyde solution (40 wt% solution) were used as solvent and reducing agent, respectively, and PVP (poly(vinylpyrrolidone)) was used as the surfactant and dispersant. Benefitting from the synergistic electronic effect between the Iridium and Cupric atoms from the alloy, the as-prepared IrCux catalysts exhibited excellent catalytic activity towards oxygen evolution reaction in 0.1 M HClO_4_ electrolyte when compared to that of commercial Ir/C catalysts, and, among them, to reach 10 mA cm^−2^, IrCu_0.77_ microspheres only required an overpotential of 282 mV, which was much lower than that of commercial Ir/C catalysts.

## 2. Experimental Section

### 2.1. Chemicals and Materials

Iridium(III) chloride (IrCl_3_, 99.8%), Copper(II) chloride dehydrate (CuCl_2_·2H_2_O, 99.9%), and Commercial Ir/C (20 wt% Ir) were purchased from Shanghai Yien Chemical Technology Co. Ltd., Shanghai, China. Ethylene glycol ((CH_2_OH)_2_, 98%), N,N-dimethylformamide (DMF, 99.5%) and Nafion solution (5 wt% solution) were obtained from Shanghai Macklin Biochemical Co. Ltd., Shanghai, China, and formaldehyde solution (HCHO, 40 wt% solution) and Poly(vinylpyrrolidone) (PVP K30, Mr ≈ 10,000) were acquired from Sinopharm Chemical Reagent Co. Ltd., Shanghai, China. Ultrapure water (Millipore water, 18.2 MΩ cm) was used in all of the experiments.

### 2.2. Synthesis of IrCu_x_ Microspheres (IrCu_0.52_, IrCu_0.77_, and IrCu_2.34_ Microspheres)

In a typical procedure of synthesis, 10 mg of IrCl_3_, 85 mg of CuCl_2_·2H_2_O, 46 mg of PVP, and 0.3 mL of HCHO (40 wt% solution) were put into 8 mL of ethylene glycol, followed by ultra-sonicating for 20 min. at 25 °C. The homogeneous blue-green solution obtained was transferred into a 25 mL Teflon reaction kettle with stainless steel reaction sleeve. The reactor was then kept at 200 °C for 5 h and it was then cooled down to 25 °C at room temperature. The heating rate of the reactor was 6 °C min.^−1^. The products were first separated by centrifugation for 30 min. at 11,000 rpm and then they were further purified by acetone/ethanol mixture for three times, and finally they were purified with ultrapure water two times. The synthesis of IrCu_0.52_ and IrCu_2.34_ microspheres were similar to the preparation of IrCu_0.77_, except that 78 and 127 mg of CuCl_2_·2H_2_O were used for IrCu_0.52_ and IrCu_2.34_, respectively.

### 2.3. Preparations of IrCu_x_/C Catalysts for Electrochemical Characterizations

1 mg of the IrCu_x_ microspheres were dispersed in 5 mL ethanol suspension containing 4 mg carbon support (Vulcan XC-72) and then sonicated for 1 h. The collection of carbon supported IrCu_x_ microspheres was fulfilled through centrifugation (11,000 rpm for 10 min.) and the products were then washed two times with ethanol in order to remove any possible impurities. The resulting carbon supported IrCu_x_ microspheres were hold at 250 °C in air for 1 h to remove the surfactants, and then they were washed several times by ethanol in order to remove calcined organic residue.

### 2.4. Physical Characterization

The characterizations of the structure and morphology of the Iridium-based catalysts samples were conducted on scanning electron microscopy (SEM, Hitachi S-4800, Tokyo, Japan) and transmission electron microscopy (TEM, JEM−2100 at 200 kV, JEOL Ltd., Tokyo, Japan). The collection of X-ray photoelectron spectra was fulfilled with the Multifunctional imaging electron spectrometer (XPS, Thermo ESCALAB 250Xi, Waltham, MA, USA).

### 2.5. Electrochemical Measurements

Before the electrochemical measurements, the IrCux microspheres were first washed with ultrapure water for several times to be used. Subsequently, the IrCux microspheres were first dispersed in ethanol suspension containing carbon support, sonicated for 1 h, and then washed with ethanol for several times. Finally, we annealed them at 250 °C in air ambiance for 1 h in order to remove excess surfactant and the microsphere could keep without obvious shape change (Figure S1). We executed the XPS experiment to investigate the chemical states of IrCux microspheres catalysts (Figure S2). The results indicated that the Iridium and Cupric elements on the surface almost transformed fully into oxidized Iridium and oxidized Cupric, respectively.

Electrochemical experiments were conducted at room temperature in a three-electrode cell. Saturated calomel electrode (SCE) was used as the reference electrode, a Pt mesh played the role of counter electrode, while a glassy carbon (GC) electrode with diameter of 3 mm was employed as the working electrode. The GC electrodes were first polished for 15 min. with Al_2_O_3_ powder with a diameter of 1.0 µm and they were washed with Millipore water and were then dried naturally before they were used. Then we started to prepare the working electrode step by step. 3 mg of IrCux/C catalysts were first dispersed in 950 μL of water and 50 μL of Nafion (5% wt) mixture solvent by ultra-sonicating for 30 min. and a homogeneous ink formed. After that, dropping 5 μL of ink onto the surface of a pre-cleaned GC electrode, dried naturally to form uniform thin film (the amount of load at 42.9 µg of IrCux microspheres/cm^2^).

All of the potentials in our experiment were versus reversible hydrogen electrode (RHE) calculated with the following equation, that is, E_RHE_ = E_SCE_ + 0.241V + 0.0591pH. The electrochemically active surface area (ECSA) was measured by testing the electrochemical double layer capacitance (Cdl). The test of cyclic voltammograms (CVs) was conducted in 0.1 M HClO_4_ solution saturated with Argon. The potential of the work electrode was swept from 0.05 V to 1.15 V at a scan rate of 100 mV s^−1^ for 10 cycles and the stable CVs were obtained. The tests of OER for all of the catalysts that were investigated in the experiment were conducted in 0.1 M HClO_4_ solution. The potential window was 1.15 V to 1.6 V and the sweep rate was 5 mV s^−1^. The current densities of OER were corrected with ohmic compensation of iR drop during the test.

## 3. Results and Discussion

### 3.1. Physical Characterization 

**Figure 1:**
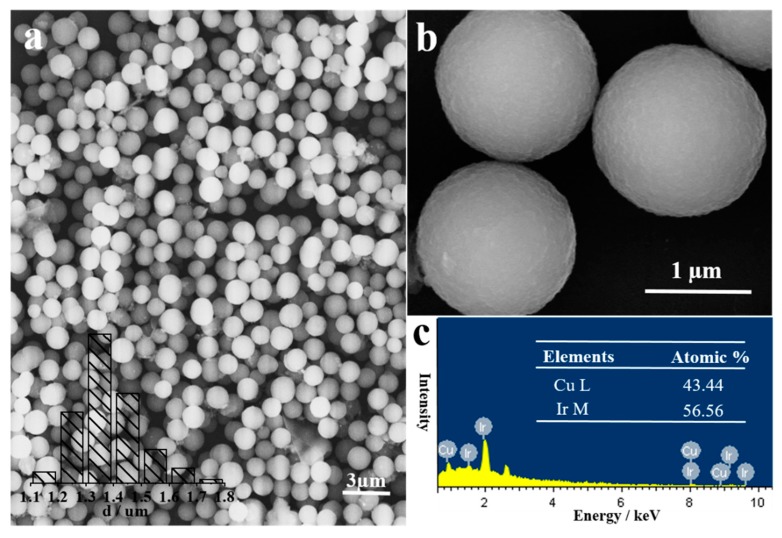
(**a**) Overview scanning electron microscopy (SEM) image of the IrCu_0.77_ microspheres and the inset was size distribution of the IrCu_0.77_ microspheres prepared using the standard synthesis, (**b**) High-magnification SEM image of the IrCu_0.77_ microspheres, (**c**) EDS of the IrCu_0.77_ microspheres, as synthesized.

Figure 1a shows a representative overview of SEM image of the synthesized IrCu_0.77_ microspheres. According to SEM image at the low-magnification, it could be found that the IrCu_0.77_ microspheres were with regular spherical shape and smooth surface. The size distribution of IrCu_0.77_ microspheres, as synthesized, was shown in the inset in Figure 1a, it could be seen that the size of IrCu_0.77_ microspheres varied from 1.1 to 1.8 µm, with an average of 1.4 µm, and relative standard deviation (RSD) = 7.9%. Figure 1b displayed a high-magnification SEM of the IrCu_0.77_ microspheres, and from it, the perfect spherical shape could also be seen, and the surfaces of the alloy microspheres were quite smooth. Figure 1c showed the energy dispersive X-Ray spectroscopy (EDS) of the synthesized sample. It could be seen that both the Cu and Ir elements were there in the microsphere and Ir/Cu atomic ratios was 56.56% to 43.44%, which we defined as IrCu_0.77_. These results indicated that high quality IrCu_0.77_ bimetallic microspheres had been successfully synthesized by the facile one-pot polyol method.

**Figure 2:**
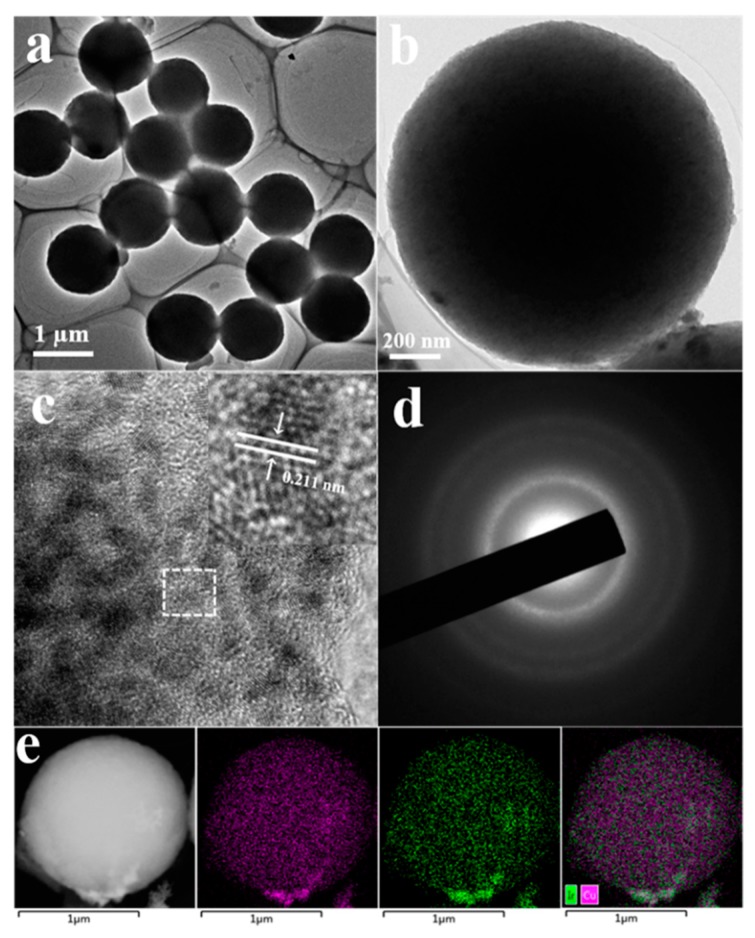
Low- (**a**) and high-magnification (**b**) TEM images of the IrCu_0.77_ microspheres prepared by standard synthesis, (**c**) High-resolution TEM (HRTEM) image of an individual IrCu_0.77_ microsphere, (**d**) Selected-area-electron diffraction (SAED) patterns of an individual IrCu_0.77_ microsphere, (**e**) HAADF-STEM image and corresponding elemental mapping of IrCu_0.77_ microsphere, Ir (green), Cu (purple).

Figure 2a showed the TEM images of the IrCu_0.77_ microspheres as-prepared at low- magnification and it could be seen that most of the samples as prepared had well sphere profile and the size of them was very uniform. Figure 2b showed the TEM images of the spherical morphology of the IrCu_0.77_ microspheres at high-magnification. The high-magnification TEM image further confirmed that the IrCu_0.77_ sample had a nice spherical shape. Figure 2c showed the high-resolution TEM (HRTEM) image of a single IrCu_0.77_ microsphere particle, and we could see that consistent and clear lattice fringes were there on the IrCu_0.77_ microspheres. The magnified HRTEM image (inset in Figure 2c) showed lattice fringes were with an inter planar spacing of 0.211 nm, which corresponded to the (111) plane of IrCu_0.77_ alloy and from the X-ray diffraction (XRD) pattern in Figure S3; it was obvious that both Ir(111) and Cu(111) peaks were there in IrCu_0.77_ microspheres and the peak position shifted a little bit from their pure metal crystal plane, which revealed the Ir-Cu alloy structure. Figure 2d showed the results of the selected area electron diffraction (SAED) pattern of an individual IrCu_0.77_ micro-sphere. As could be seen, a series of concentric circles were clearly there, which indicated the polycrystalline structure of the sample, as synthesized. The element mapping characterization was employed to check the elemental distribution of Iridium and Cupric element in the microsphere. As shown in Figure 2e, both the Iridium and Cupric elements were evenly distributed throughout the whole domain of the IrCu_0.77_ micro-sphere, which confirmed their intrinsic feature of alloy. In addition, by increasing or decreasing the amount of copper precursor used, the IrCu_2.34_ microspheres and IrCu_0.52_ microspheres could be obtained, respectively (Figures S4 and S5).

**Figure 3:**
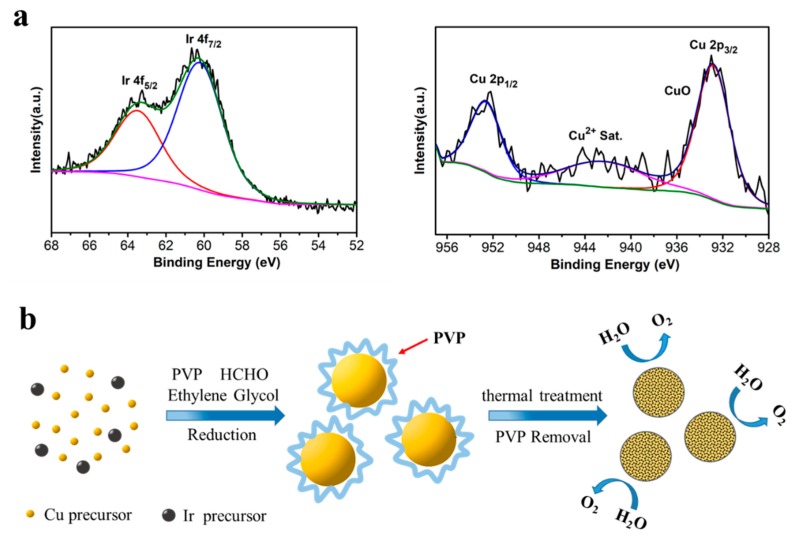
(**a**) X-ray photoelectron spectroscopy (XPS) spectra of Ir 4f and Cu 2p of IrCu_0.77_ microspheres, (**b**) Schematic illustration for the synthesis of IrCux microspheres catalysts for oxygen evolution reaction.

Figure 3a showed the XPS data of the IrCu_0.77_ microspheres, as synthesized. The Ir 4f_7/2_ peaks of IrCu_0.77_ microspheres could be assigned to Ir^0^ 4f_7/2_, Ir^0^ 4f_5/2_, and their locations were at 60.31 and 63.38 eV, respectively [27]. Among the peaks, it could be clearly observed that the Ir^0^ peaks had strong intensity, which indicated that the metallic Ir was successfully formed. Additionally, it could also be observed that the peak in the spectrum of Cu 2p_3/2_ and Cu 2p_1/2_, which were located at 932.92 eV and 952.75 eV, respectively; moreover, the satellite structure of Cu^2+^ could be seen clearly, the above results could be attributed to the characteristic peaks of Cu 2p at oxidative state [36]. The oxidative state of Cu 2p might originate from surface oxidation under the air ambiance. In comparison with the standard location of Cu 2p peak and Ir 4f peak, the Ir 4f peak of IrCu_0.77_ had positively moved about 0.29 eV, while the Cu 2p peak shifted positively to about 1.63 eV. The results further confirmed the successful synthesis of IrCu alloy microspheres, which had significant electron interactions between Iridium and Cupric elements at the atomic level [32].

Figure 3b illuminated the facile synthesis strategy of IrCu_x_ microspheres that were based on the polyol method and the organic surfactant, which strongly adsorbed on the surface of the catalysts, was effectively removed through heat treatment in air and, finally, the IrCu_x_ catalyst was applied to the OER. The polyol method was a simple, green, and economic synthesis method, and we could facile synthesize a large number of high quality catalysts through this facile and efficient green synthesis method. The polyol method used ethylene glycol as solvent and reaction medium, which had many advantages in the synthesis of crystals. For example, ethylene glycol had outstanding solubility to inorganic salts, and as an excellent solvent, ethylene glycol facilitated diffusion growth control of crystals, so that the product size was uniform; in addition, ethylene glycol had a certain degree of reducibility, which facilitated the rapid decomposition of the precursor. In the typical process of synthesis, the IrCu_x_ microspheres could be prepared through the facile polyol method. In the process, the Iridium(III) chloride and Copper(II) chloride dehydrate were employed as the metal precursors, while formaldehyde solution (40 wt% solution) and ethylene glycol were employed as reducing agent and solvent respectively.PVP was used as the surfactant and dispersant, and the resulting microspheres were seriously agglomerated in the case of not adding PVP during the synthesis process (Figure S6). To study the key factors for the successful synthesis of IrCu microspheres, a series of experiments for comparison were designed and conducted. All other conditions remained the same, and the quantity of formaldehyde solution changed from 0.3 mL either to 0.15 mL or to 0.6 mL, the particle size was significantly reduced, but it still kept the spherical morphology (Figure S7). The change in the amount of formaldehyde affected the rate of reduction of the system, thereby affecting the size of the products. When other common solvents replaced ethylene glycol (such as water, DMF), particles with a spherical morphology could no longer be obtained, instead, some particles with morphologies other than specific shape formed (Figure S8). It indicated that ethylene glycol promoted the formation of spherical morphology, guiding the crystals to develop into the shape of the sphere during the process of growth. Furthermore, we checked the effect of the reaction temperature. If we reduced the reaction temperature to 180 °C, the products were mainly small spherical particles. If the temperature increased to 220 °C, the products were mainly crystals with irregular morphologies (Figure S9). Through the series of temperature comparative experiments, we could find that proper temperature control was quite essential in the successful synthesis of IrCu microspheres with high-quality. To make an insight into the evolution of morphology of IrCu microspheres, we also carried out time sequential evolution experiments in the hydrothermal process. The reaction time was extended from 5 h to 8 h, and the morphology and particle size of the IrCu microspheres did not change significantly (Figure S10). Moreover, without the addition of the Ir precursor, we could obtain copper nanospheres with different sizes (Figure S11).

### 3.2. Oxygen Evolution Reaction

**Figure 4:**
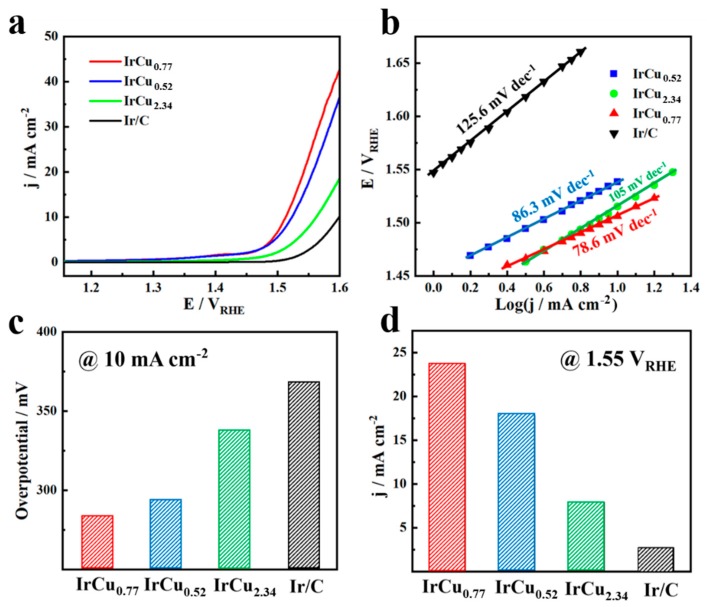
Electrochemical characterization of IrCu_0.77_ microspheres versus IrCu_0.52_ microspheres, IrCu_2.34_ microspheres and commercial Ir/C as oxygen evolution reaction (OER) catalysts. (**a**) Polarization curves, (**b**) Tafel plots. Bar graph showing the (**c**) overpotential at 10 mA cm^−2^, and (**d**) current densities at the potential of 1.55 V_RHE_. All catalysts were examined using 0.1 M HClO_4_ as the electrolyte at the scan rate of 5 mV s^−1.^

Figure 4a showed the polarization curves of OER of IrCux microspheres and commercial Ir/C catalysts in 0.1M HClO_4_ solution and the scan rate was 5 mV s^−1^. The IrCux microspheres exhibited enhanced OER catalytic activities when compared to commercial Ir/C catalysts. The onset potentials of IrCu_0.77_ and IrCu_0.52_ microspheres were at 1.47 V_RHE_, which were lower than that of the IrCu_2.34_ (1.51 V_RHE_), while the onset potential of the commercial Ir/C was higher at 1.55 V_RHE_. Figure 4b showed the corresponding tafel slope of different catalysts. In comparison to that of commercial Ir/C, the IrCux microspheres also showed much lower tafel slope, which further confirmed their faster reaction kinetics. This result, combined with the results from EDS, XPS, and XRD, indicated that the enhancement of the catalytic activities of IrCux microspheres could mainly originate from the synergistic electronic effect between the Iridium and Cupric atoms from the alloy. As previous literatures reported, the interaction between Ir and Cu atoms could modify the electronic structure (d-band center) of the Ir atoms, and therefore improved their catalytic activities towards OER [22,30,41]. Figure 4c showed the comparison of overpotentials for different catalysts at 10 mA cm^−2^, that was, the IrCu_0.77_ microspheres only needed an overpotential of 282 mV and this was quite lower than those of the IrCu_0.52_ microspheres (293 mV), IrCu_2.34_ microspheres (337 mV), and Ir/C (367 mV). Figure 4d showed the comparison of current densities for the different catalysts samples at 1.55 V_RHE_, and the activities were in the following order of IrCu_0.77_ microspheres > IrCu_0.52_ microspheres > IrCu_2.34_ microspheres > commercial Ir/C.

**Figure 5:**
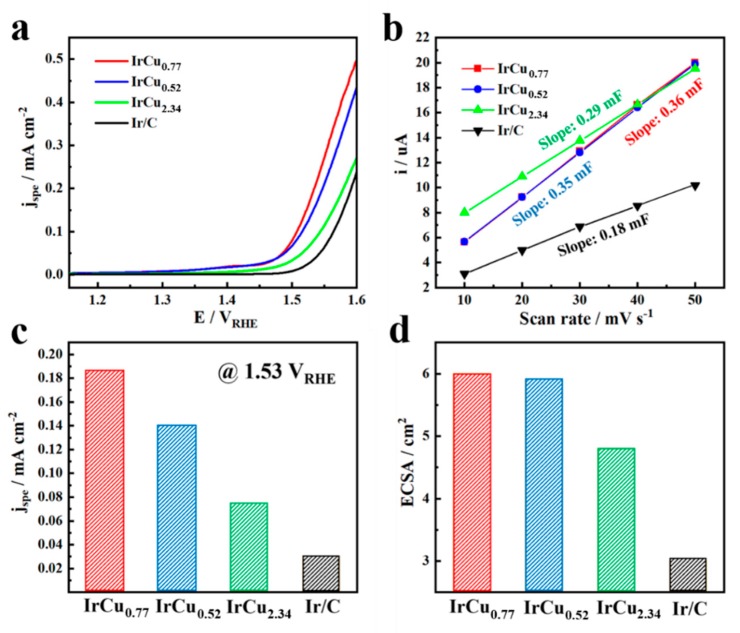
(**a**) Electrochemical surface area (ECSA)-corrected polarization curves of IrCu_0.77_ microspheres versus IrCu_0.52_ microspheres, IrCu_2.34_ microspheres and commercial Ir/C catalysts. (**b**) Charging currents measured at 0.86 V_RHE_ or 0.9 V_RHE_ plotted as a function of scan rate. The double layer capacitance of the system was taken from the slopes of the linear fit to the data. Bar graph showing the (**c**) current densities normalized to the electrochemically active surface area at OER overpotential of 300 mV (1.53 V_RHE_) and (**d**) ECSAs of different catalysts.

For the purpose of representing the intrinsic activity of catalysts, we explored the IrCux microspheres electrochemical surface areas (ECSAs) by testing the electrochemical double layer capacitance (Figure S12). According to related research, the surface electric double layer capacitance of the ideal smooth oxide was 60 µF cm^−2^ [42]. Figure 5a,c showed the ECSA-corrected polarization curves and current densities (@1.53 V_RHE_) of different catalysts, respectively, and it could be found that the specific activities of IrCu_0.77_ microspheres showed significant enhancement in comparison to those of the IrCu_0.52_ microspheres, IrCu_2.34_ microspheres, and commercial Ir/C catalysts. Figure 5b,d showed the double layer capacitance slopes and the corresponding electrochemical surface areas of different catalysts, respectively. Obviously, the IrCu_0.77_ microspheres and IrCu_0.52_ microspheres exhibited increased ECSAs as compared to those of IrCu_2.34_ microspheres and commercial Ir/C catalysts. Overall, the above researches showed that the specific activity of IrCu_0.77_ microspheres was the highest, and the results further confirmed their excellent electrocatalytic performance for OER in acidic condition.

## 4. Conclusions

In conclusion, we have presented a facile one-pot synthesis that is based on polyol method for preparing IrCu microspheres and through this facile and efficient polyol method of synthesis, we obtained a large number of well-formed IrCu microspheres with uniform size and good dispersion successfully. In the typical procedure of synthesis, formaldehyde solution and ethylene glycol were employed as reducing agent and solvent, respectively, while PVP were used as surfactant and dispersant, and all of them played indispensable roles for the successful synthesis of alloy Ir-Cu microspheres with uniform size and smooth surface. IrCu_0.77_ microspheres exhibited outstanding catalytic activity towards OER in 0.1 M HClO_4_ electrolyte due to the synergistic electronic effect between the Iridium and Cupric atoms from the alloy. Additionally, IrCu_0.77_ microspheres only required an overpotential of 282 mV to achieve 10 mA cm^−2^, and this was significantly lower than that of commercial Ir/C catalysts (367 mV), representing an outstanding Ir-based OER electrocatalysts via the polyol method. Since the microsphere catalysts, as synthesized, had the size scale between nanoscale and bulk catalysts, they might play a transition role of bridge to study the size effect on electrocatalytic performance from bulk single crystalline plane to nanocatalysts.

## Supplementary Materials

The following are available online at https://www.mdpi.com/2079-4991/9/8/1145/s1, Figure S1: Low- (a) and high-magnification (b) SEM images of IrCu_0.77_/C catalysts after thermal treatment; Figure S2: XPS spectra of the (a) Ir 4f and (b) Cu 2p peaks of IrCu_0.77_/C catalysts after thermal treatment; Figure S3: XRD pattern of IrCu_0.77_ microspheres; Figure S4: (a) Overview SEM image of the IrCu_2.34_ microspheres and the inset was size distribution of the IrCu_2.34_ microspheres prepared using the standard synthesis, (b) High-magnification SEM image of the IrCu_2.34_ microspheres, (c) EDS of the IrCu_2.34_ microspheres as synthesized; Figure S5: (a) Overview SEM image of the IrCu_0.52_ microspheres and the inset was size distribution of the IrCu_0.52_ microspheres prepared using the standard synthesis, (b) High-magnification SEM image of the IrCu_0.52_ microspheres, (c) EDS of the IrCu_0.52_ microspheres as synthesized; Figure S6: SEM images of IrCu microspheres without PVP in the standard system for the synthesis of IrCu_0.77_ microspheres; Figure S7: SEM images of IrCu_0.77_ microspheres with different amount of formaldehyde solution (a) 0.15 mL and (b) 0.6 mL; Figure S8: SEM images of IrCu nanoparticles collected from the reactions with the same conditions used in the synthesis of monodispersed IrCu_0.77_ microspheres (Figure 1a) but with ethylene glycol replaced by (a) H2O and (b) DMF; Figure S9: SEM images of IrCu_0.77_ microspheres with different reaction temperature (a) 180 °C and (b) 220 °C; Figure S10: SEM images of the samples obtained at various reaction times for a standard IrCu_0.77_ microspheres synthesis: (a) 5.0, (b) 6.0, (c) 7.0, and (d) 8 h, respectively; Figure S11: SEM images of Cu microspheres without Ir precursor in the standard system for the synthesis of IrCu_0.77_ microspheres; Figure S12: Charging currents measured at the non-faradaic potential of 0.5 V–0.65V or 0.55 V–0.65 V (vs. RHE) at different scan rates (10, 20, 30, 40 and 50 mV s^−1^) for IrCu_0.77_ microspheres (a), IrCu_0.52_ (b), IrCu_2.34_ (c) and Ir/C (d).
