# Supplementary material for: Facile Synthesis of IrCu Microspheres Based on Polyol Method and Study on Their Electro-Catalytic Performances to Oxygen Evolution Reaction
Source: Nanomaterials (Basel). 2019 Aug 10;9(8):1145. doi: 10.3390/nano9081145 (PMC6724051; doi:10.3390/nano9081145)
Supplement: Supplementary file 1 [file nanomaterials-09-01145-s001.pdf]

Article

Facile Synthesis of IrCu Microspheres Based on Polyol Method and Study on Their Electro-Catalytic Performances to Oxygen Evolution Reaction

Xuan Liu [1,†], Zichao Li [2,†], Luming Zhou [1], Kuankuan Wang [1], Xihui Zhao [1], and Qun Li [1], Yujia Deng [1,*]

[1] School of Chemistry and Chemical Engineering, Qingdao University, Qingdao 266071, China
[2] College of Life Sciences, Qingdao University, Qingdao 266071, China
* Correspondence author: dengyujia@qdu.edu.cn
† These authors contributed equally to this work.

Received: 5 July 2019; Accepted: 8 August 2019; Published: date

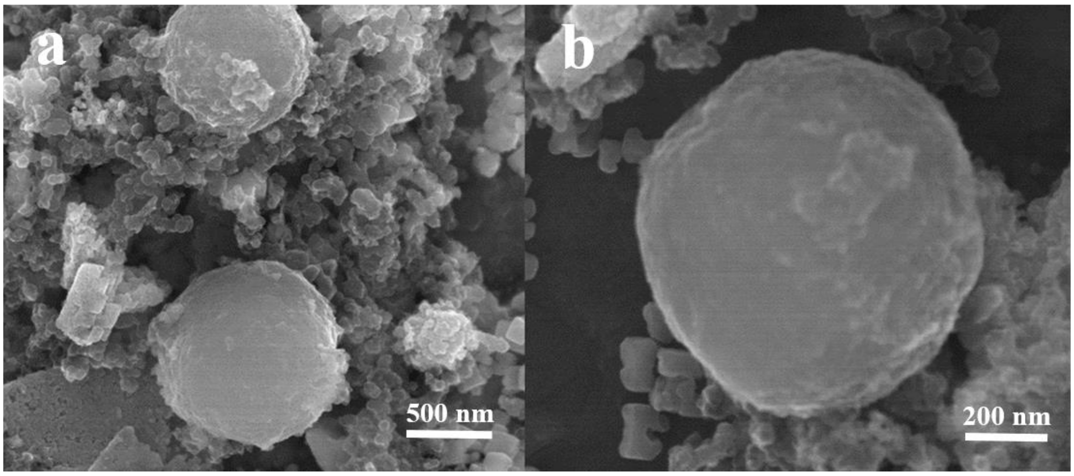

Figure S1. Low- (**a**) and high-magnification (**b**) SEM images of $IrCu_{0.77}/C$ catalysts after thermal treatment.

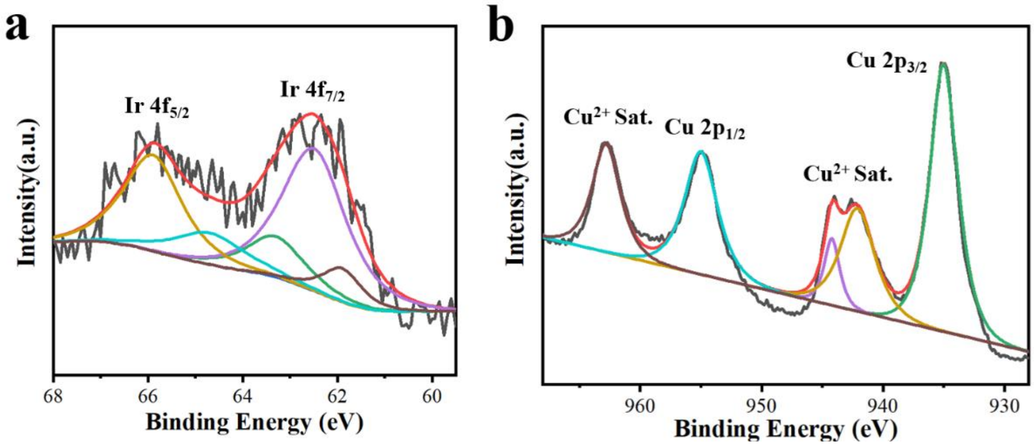

Figure S2. XPS spectra of the (**a**) Ir 4f and (**b**) Cu 2p peaks of $IrCu_{0.77}/C$ catalysts after thermal treatment.

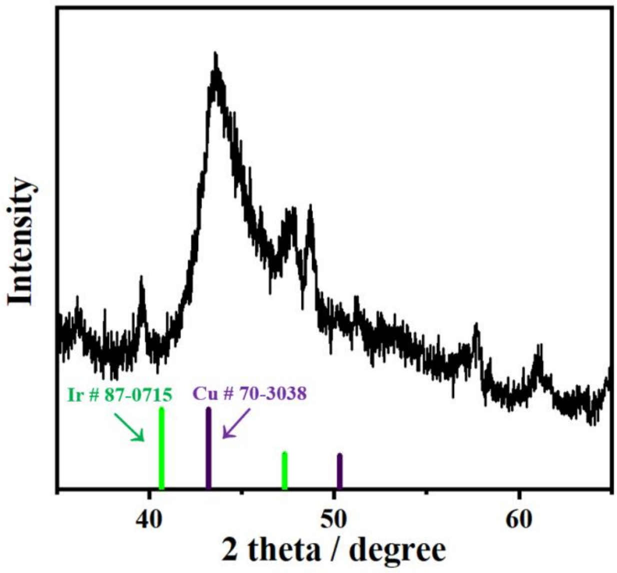

Figure S3. XRD pattern of $IrCu_{0.77}$ microspheres.

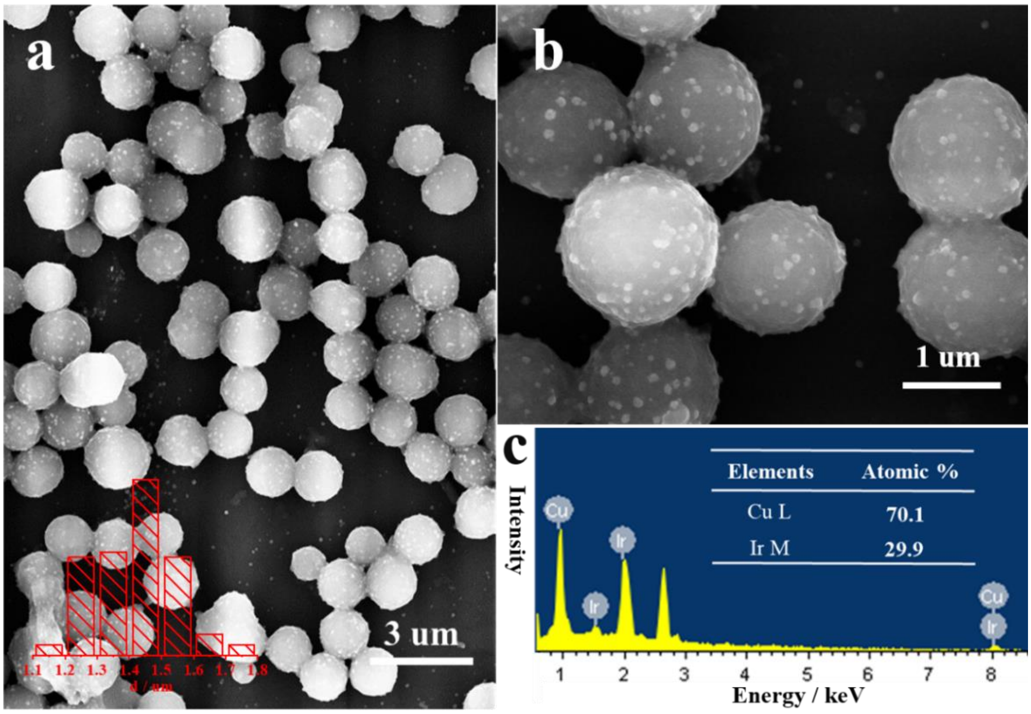

Figure S4. (**a**) Overview SEM image of the $IrCu_{2.34}$ microspheres and the inset was size distribution of the $IrCu_{2.34}$ microspheres prepared using the standard synthesis, (**b**) High-magnification SEM image of the $IrCu_{2.34}$ microspheres, (**c**) EDS of the $IrCu_{2.34}$ microspheres as synthesized.

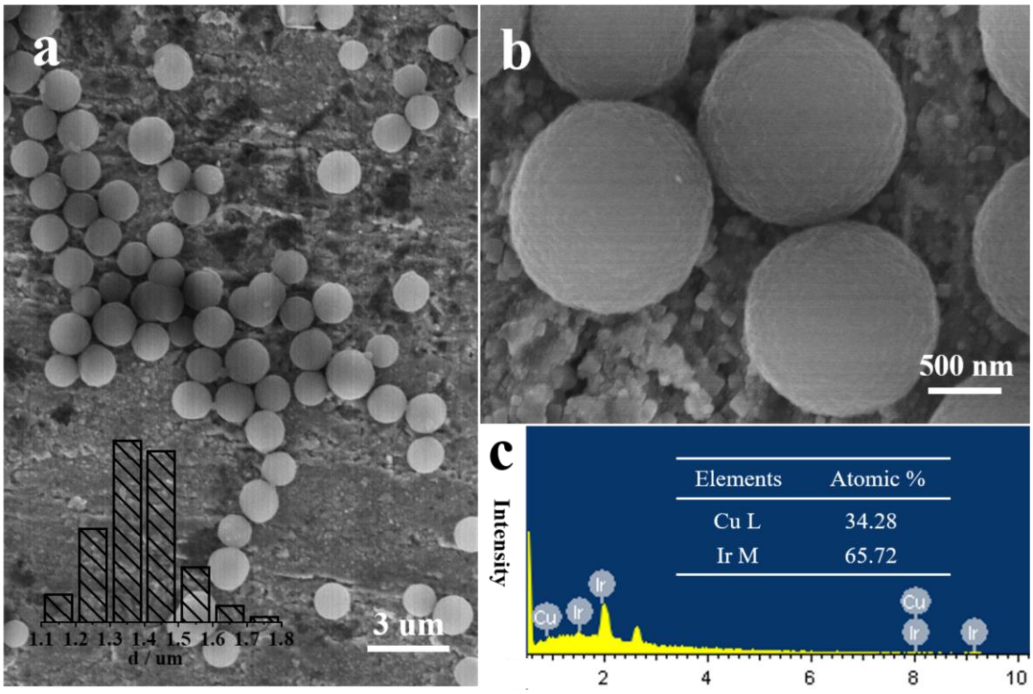

Figure S5. (**a**) Overview SEM image of the $IrCu_{0.52}$ microspheres and the inset was size distribution of the $IrCu_{0.52}$ microspheres prepared using the standard synthesis, (**b**) High-magnification SEM image of the $IrCu_{0.52}$ microspheres, (**c**) EDS of the $IrCu_{0.52}$ microspheres as synthesized.

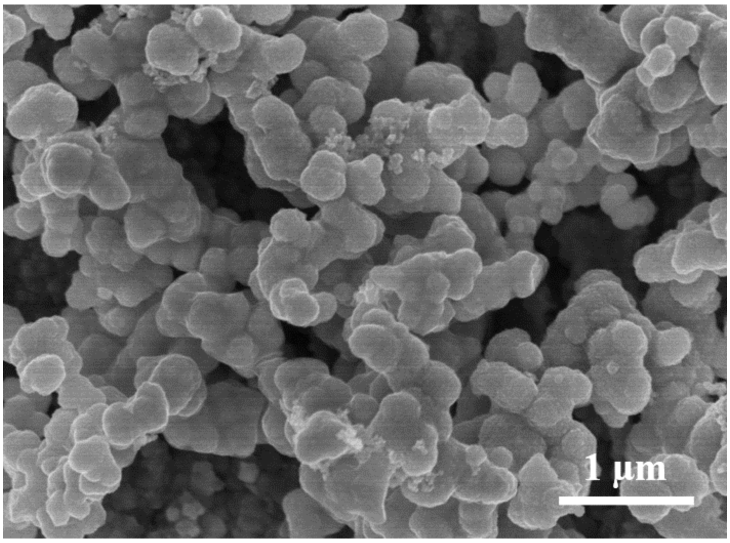

Figure S6. SEM images of IrCu microspheres without PVP in the standard system for the synthesis of $IrCu_{0.77}$ microspheres.

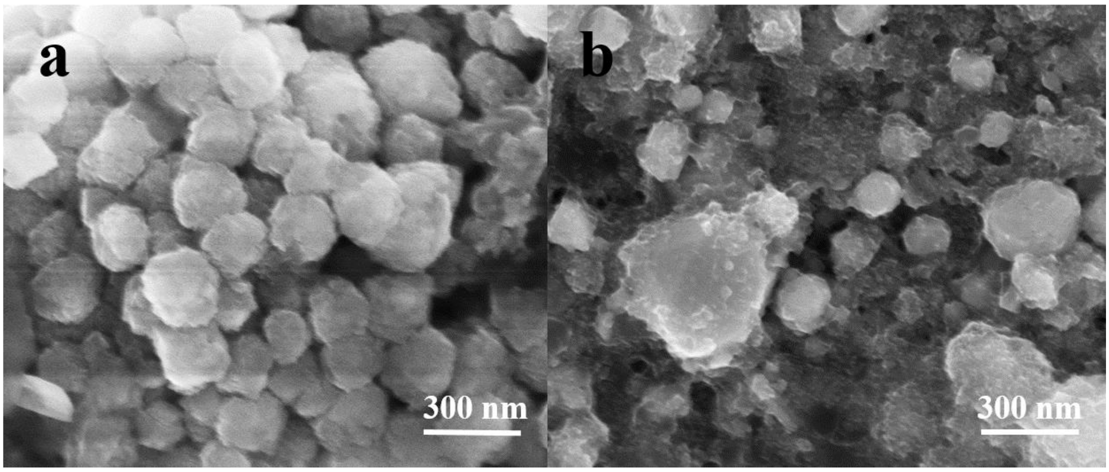

Figure S7. SEM images of $IrCu_{0.77}$ microspheres with different amount of formaldehyde solution (**a**) 0.15 mL and (**b**) 0.6 mL.

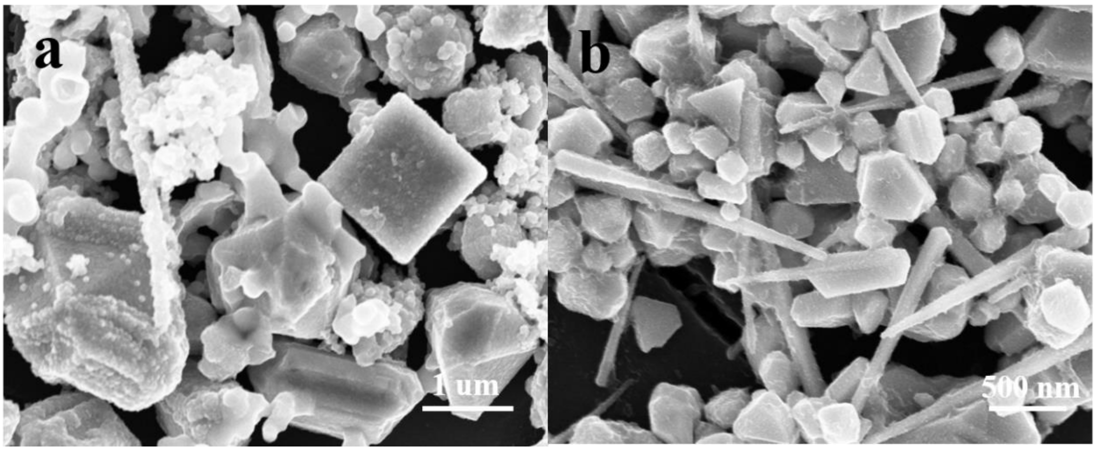

Figure S8. SEM images of IrCu nanoparticles collected from the reactions with the same conditions used in the synthesis of monodispersed $IrCu_{0.77}$ microspheres (Figure 1a) but with ethylene glycol replaced by (**a**) H_2O and (**b**) DMF.

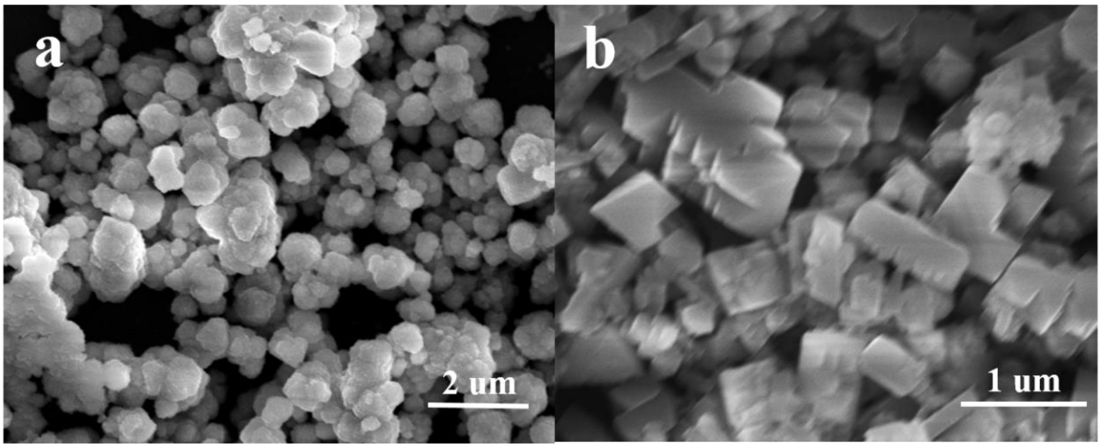

Figure S9. SEM images of $IrCu_{0.77}$ microspheres with different reaction temperature (**a**) 180℃ and (**b**) 220 °C.

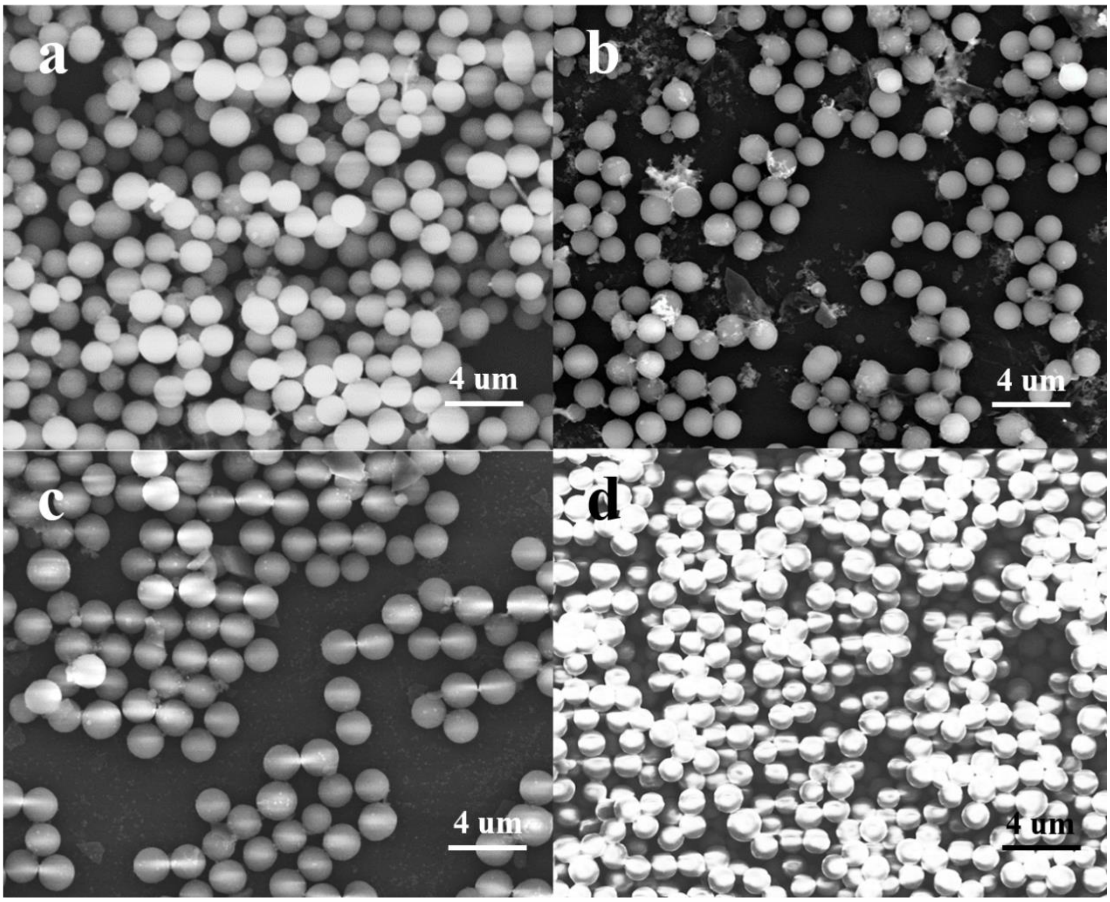

Figure S10. SEM images of the samples obtained at various reaction times for a standard $IrCu_{0.77}$ microspheres synthesis: (**a**) 5.0, (**b**) 6.0, (**c**) 7.0, and (**d**) 8 h, respectively.

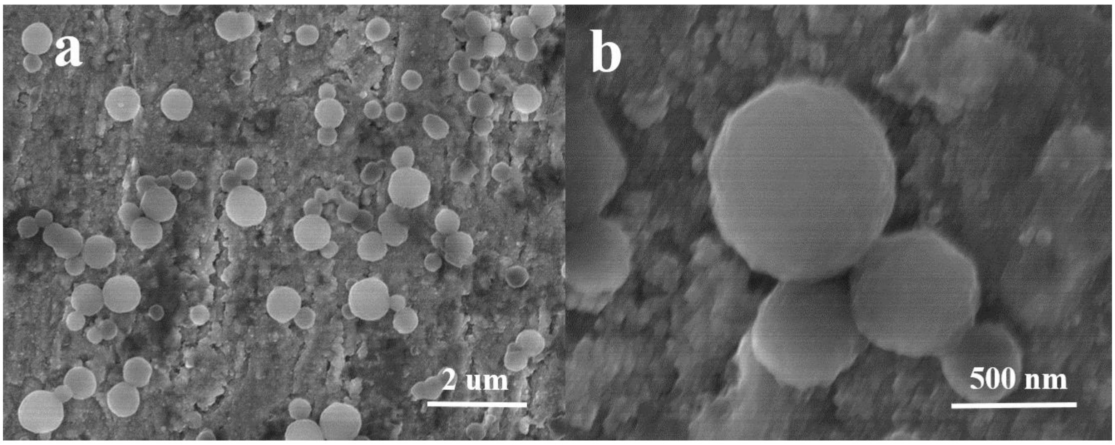

Figure S11. SEM images of Cu microspheres without Ir precursor in the standard system for the synthesis of $IrCu_{0.77}$ microspheres.

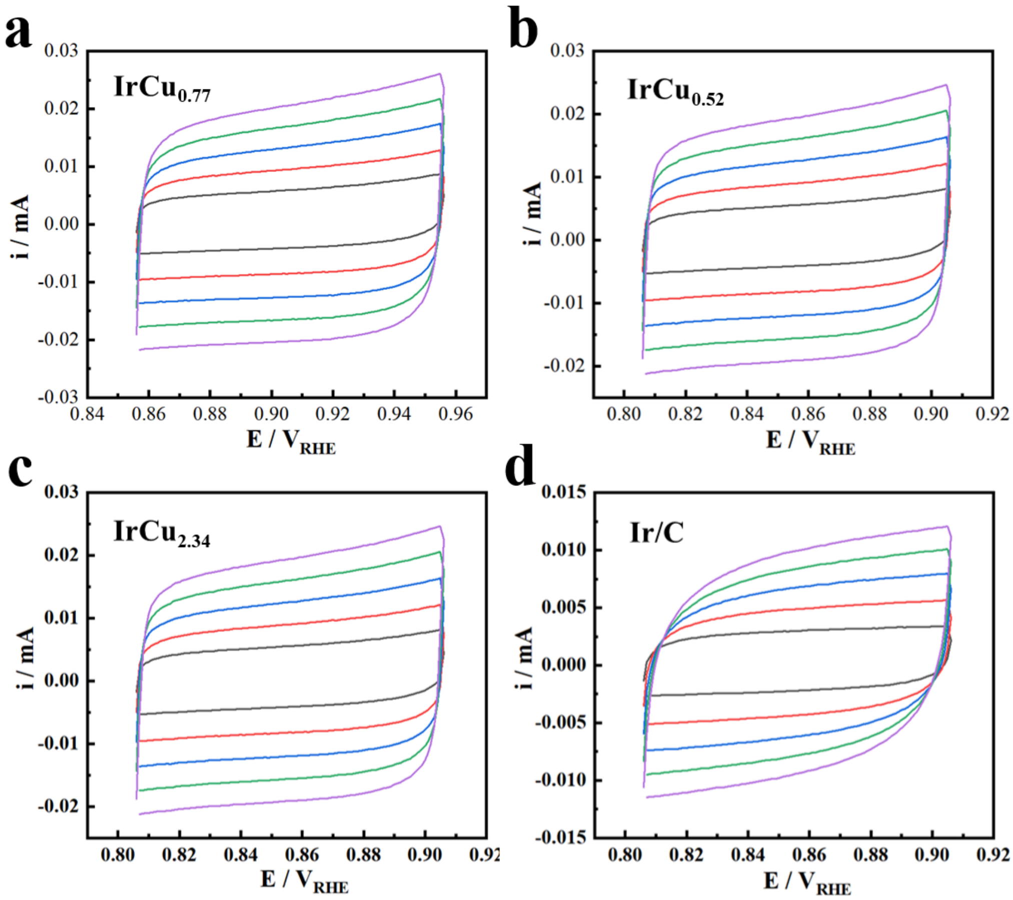

Figure S12. Charging currents measured at the non-faradaic potential of 0.5 V–0.65V or 0.55 V–0.65 V (vs. RHE) at different scan rates (10, 20, 30, 40 and 50 mV s^{-1}) for $IrCu_{0.77}$ microspheres (**a**), $IrCu_{0.52}$ (**b**), $IrCu_{2.34}$ (**c**) and Ir/C (**d**).
